# Mechanical and Electrical Phenotype of hiPSC‐Cardiomyocytes on Fibronectin‐Based Hydrogels

**DOI:** 10.1002/adhm.202501595

**Published:** 2025-11-29

**Authors:** Ana Da Silva Costa, Lineta Stonkute, Sara Trujillo, Mariana Azevedo Gonzalez Oliva, Francis Burton, Matthew J. Dalby, Oana Dobre, Godfrey Smith, Manuel Salmeron‐Sanchez

**Affiliations:** ^1^ School of Cardiovascular and Metabolic Health University of Glasgow Glasgow UK; ^2^ MVLS Graduate School University of Glasgow Glasgow UK; ^3^ Centre for the Cellular Microenvironment University of Glasgow Glasgow UK; ^4^ Institute for Bioengineering of Catalonia (IBEC) The Barcelona Institute for Science and Technology (BIST) Barcelona Spain; ^5^ Institució Catalana de Recerca i Estudis Avançats (ICREA) Barcelona Spain

**Keywords:** hydrogels, iPSC‐cardiomyocytes, mechanical properties

## Abstract

A major challenge in cardiac research is the limited translatability of drug screening and toxicity assays due to the use of in vitro models that poorly mimic the native cardiac environment. Human induced pluripotent stem cell‐derived cardiomyocytes (hiPSC‐CMs) offer a promising route forward, but conventional 2D culture on rigid substrates hinders their functional maturation and predictive accuracy. This study addresses this problem by investigating the effect of hybrid fibronectin‐based hydrogels with tunable stiffness on the mechanical and electrical properties of hiPSC‐CMs. We engineered hydrogels with stiffness mimicking the lowest range of neonatal heart tissue stiffness (2–4 kPa) and compared hiPSC‐CM behavior on these substrates to that on standard fibronectin‐coated glass. Our results demonstrate that hydrogel culture promotes more uniform and stable cardiomyocyte contractions, as evidenced by increased single peak percentages and altered contraction duration. Electrophysiological analysis revealed that hydrogel stiffness influences action potential duration and signal amplitude. Furthermore, hiPSC‐CMs on hydrogels exhibited enhanced cell‐matrix and cell–cell adhesion, indicating improved structural and functional connectivity. Drug testing with known cardioactive compounds, including isoproterenol and nifedipine, revealed distinct differences in drug responses between hydrogel and glass cultures, suggesting that hydrogels provide a more physiologically relevant platform for assessing drug effects. This work highlights the potential of engineered hydrogel substrates to enhance the functional maturity and predictive accuracy of hiPSC‐CMs for cardiac research and drug development.

## Graphical abstract

We introduce fibronectin‐based PEG hydrogels with controlled rigidity to enable the culture of iPSC‐derived cardiomyocytes. These substrates offer an alternative to the current culture of these cells on fibronectin‐coated glass, providing enhanced structural and functional behavior. The system provides a more physiologically relevant platform to assess drug cardiotoxicity.

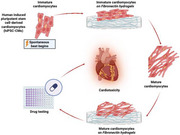

## 1 Introduction

Cardiac regenerative medicine, as well as drug and toxicology screenings, require suitable cells to compensate for the loss of cardiomyocyte function in the diseased heart [1], and for personalized drug screening with relevant disease models [2, 3]. The first generation of induced pluripotent stem cells (iPSCs) [4] in 2006 was a significant breakthrough, which allowed the production of human stem cells in vitro without ethical constraints. These can be differentiated into any cell type, including cardiomyocytes [2].

Between 1988 and 2009, 14 drugs were removed from the market due to their potential to induce life‐threatening cardiac arrhythmias [5, 6, 7]. hiPSC‐cardiomyocytes (hiPSC‐CMs) have been shown to have an electrophysiology that responds to a range of clinically well‐characterized compounds in a manner that reliably predicts their effect on the adult human heart [8]. This feature makes hiPSC‐CMs a strong candidate for low‐cost in vitro cardiotoxicity screening.

This study was designed to investigate the effect of substrates of different mechanical stiffness on the contractile behavior of the cardiomyocytes, with the aim of elucidating the role of stiffness in the context of a screening assay for toxicology/lead development in both academic and commercial labs. Tissue engineering aims to develop in vitro living tissues using biomaterials and biochemical factors [9, 10]. The substrate on which hiPSC‐CMs are cultured plays an important role in their function. The stiffness of a neonatal heart is in the range of 3.1–13.5 kPa [11, 12], and yet cells are typically cultured on plastic or glass that have very unphysiological stiffness [13, 14].

Studies have shown that cardiomyocytes are highly dependent upon the load against which the cell is contracting [11, 15, 16]. For example, the force of spontaneous CM contraction was shown to decrease with increasing substrate stiffness, and this force is similar to that of neonatal rat cardiomyocytes [11].

In the context of regenerative medicine and tissue engineering, achieving mature cardiomyocytes is essential for developing effective heart disease therapies [17]. The extracellular matrix (ECM) plays a pivotal role in this maturation process by providing the necessary biochemical and mechanical cues that guide cardiomyocyte development and integration into engineered tissues [18]. Cardiomyocyte maturation depends not only on mechanical properties but also on biochemical signals from the ECM [19]. The composition and architecture of ECM hydrogels can modulate cellular behavior, affecting gene expression, ion channel activity, and contractile function [20]. Studies have demonstrated that incorporating specific growth factors or peptides within hydrogels can enhance electrical coupling between hiPSC‐CMs, promoting more mature electrophysiological characteristics similar to adult cardiomyocytes [21]. Furthermore, advanced biophysical techniques like optogenetics offer promising ways to investigate how varying stiffness and biochemical environments affect cardiac cell functionality at the molecular level [22]. This multifaceted approach to understanding cardiomyocyte maturation may lead to improved strategies for both regenerative therapies and drug screening protocols, ultimately resulting in safer and more effective treatments for heart diseases. By utilizing these innovative methodologies, researchers can gain deeper insights into cardiac development mechanisms while creating more robust in vitro models that closely mimic the native heart environment [23].

Here, we investigate the mechanical and electrophysiological properties of hiPSC‐CMs cultured on hybrid fibronectin‐based hydrogels with varying stiffnesses (∼2.2 and 4.4 kPa). We assess the viability of these iPSC‐CMs on hydrogels for cardiotoxicity screening by evaluating their responses to well‐established pharmacological agents, focusing on both electrophysiological parameters and cellular motility.

Recent advancements in cardiac tissue engineering have demonstrated the potential of the 3D Engineered Heart Tissues and platforms like BioWire for achieving maximal structural and functional maturation [24, 25, 26]. These 3D systems are crucial for achieving maximal structural and functional maturation over extended culture periods (often 6–8 weeks) [27], and they have demonstrated utility in industrial drug screening settings. However, our study employs a flexible 2D monolayer approach specifically optimized for high‐throughput screening, where certain operational requirements take precedence over maximal tissue complexity. The monolayer format is critical for: (1) enabling high‐resolution optical access necessary for accurate spatiotemporal analysis of contraction using algorithms like MUSCLEMOTION [28]; (2) ensuring reliable pharmacokinetics by guaranteeing cells are exposed to a known, free concentration of drug in the aqueous medium, which is less certain in porous 3D gels due to partitioning effects [29]; and (3) maintaining compatibility with existing high‐throughput screening plate formats (96‐well or higher) [28]. Our work is thus positioned to improve the predictive accuracy of primary, high‐throughput cardiotoxicity assays by introducing a physiological mechanical cue (2–4 kPa) without compromising high‐throughput screening feasibility.

## 2 Methods

### 2.1 Hydrogel Synthesis

Fibronectin (FN, YoProteins) was poly‐(ethylene glycol) (PEG)ylated using a previously published procedure [30]. PEG hydrogels were formed using an UV‐initiated polymerization using the protocol from Dobre et al. [31]. A final concentration of 1 mg mL^−1^ PEGylated FN was added to different concentrations of 4‐arm‐PEG‐Acrylate (PEGAc, 10 kDa, LaysanBio) (5 wt. % or 10 wt. %), and to 8‐arm‐PEG‐Acrylate (20 kDa, Creative PEGworks) (5 wt. %). A protease‐degradable peptide, flanked by two cysteine residues (VPM peptide, GCRDVPMSMRGGD‐RCG, purity 96.9%, Mw 1696.96 Da, GenScript) was added at a final concentration of 40 mg mL^−1^. The UV initiator, Irgacure (2‐Hydroxy‐4′‐(2‐hydroxyethoxy)‐2‐methylpropiophenone, Sigma–Aldrich) was added to the mixture of PEGylated FN, PEG‐Ac, and VPM peptide at a final concentration of 0.05 wt. % prior to UV irradiation. A hydrogels chemistry is shown in Figure S1.

All reagents were dissolved in Dulbecco's phosphate‐buffered saline (DPBS, no calcium, no magnesium, ThermoFisher). The mixtures were transferred to 35 mm glass‐bottom petri dishes (MatTek) with a diameter of 5 mm Polydimethylsiloxane (PDMS) mold. A hydrophobic glass coverslip was placed on top to seal the samples and create a flat surface. Samples were curated under a UV lamp (Excellitas Omnicure S1500, filter 320–390 nm) at 10 mW cm^−2^ for 10min. The coverslip and mold were removed, and the hydrogels were kept in PBS ++ either overnight at 4 °C or at 37 °C for less than 3 h. In the case that cells were embedded in the hydrogel, they were added just before UV irradiation (Figure S2). Where cells were under the hydrogel, the cell culture was performed using a diameter of 3 mm PDMS stencil, on previously fibronectin‐coated glass (10 µg mL^−1^). Cell cultures settled overnight to adhere to the substrate before the hydrogel was added and crosslinked onto the cell patch formed (Figure S2). Where cells were added onto gels, this was performed after crosslinking was complete. For the 96‐well format, hydrogel samples were loaded onto 96‐well, glass‐bottom plates (MatTek) to a final depth of 1 mm. The hydrogels in the wells were then cured as before under a UV lamp.

### 2.2 Cell Culture

**FIGURE 1:**
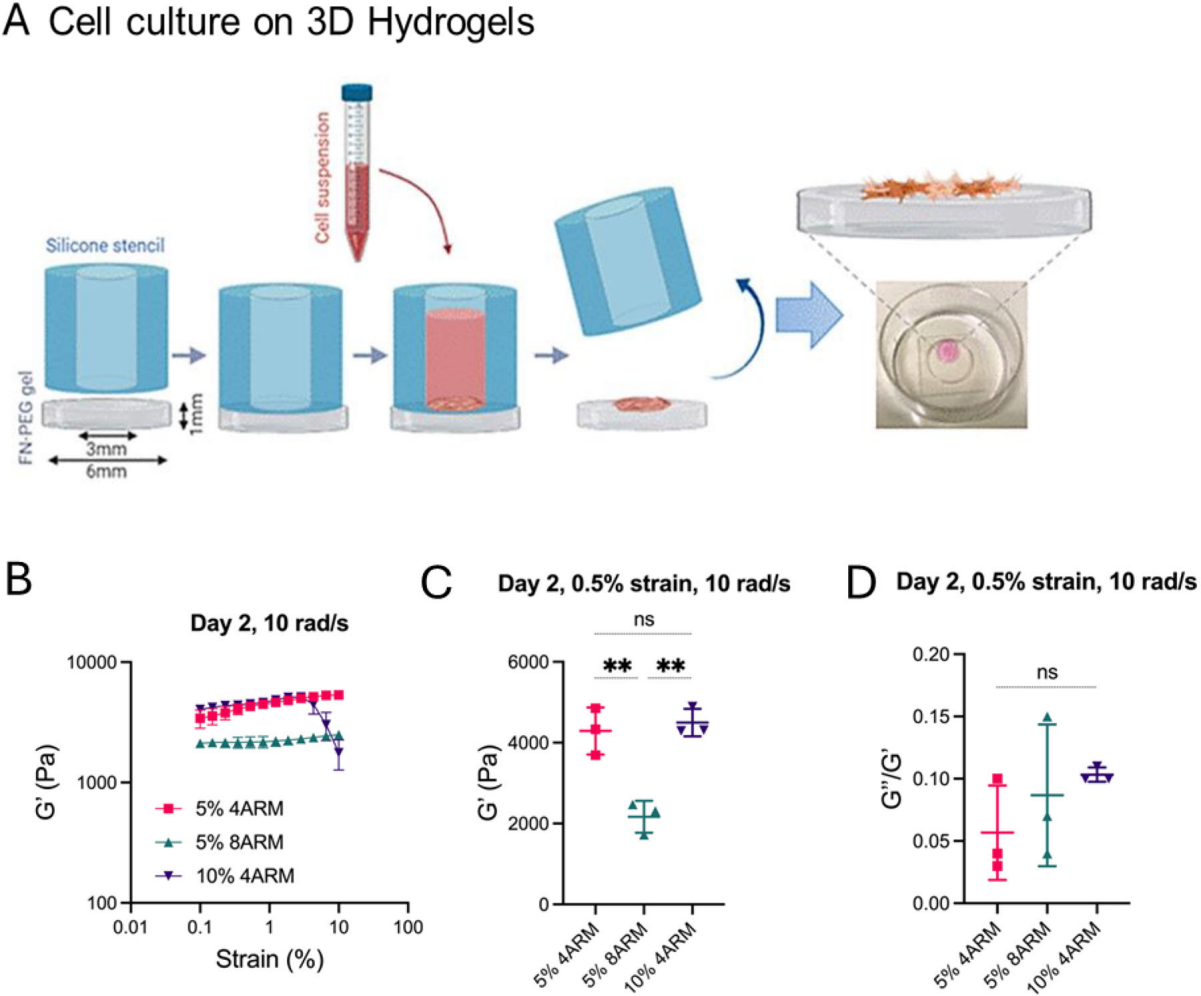
Methodology for cultures on hydrogel and stiffness measurements. (A) FN‐PEG gels are curated using 6 mm PDMS molds. A silicone stencil is deposited onto the hydrogel through which hiPSC‐CMs are inserted. After 48 h, the silicone stencil is removed, and fresh cell culture medium is added until the cell culture is covered;(B) Shear storage modulus (*G′*) at 10 rad/s of all hydrogel conditions plotted as a function of strain. Plotted as mean ± SEM. Shear storage modulus (*G′*) (C) and Loss modulus(G″)/storage modulus(*G′*) or Tan δ(D) of all hydrogel conditions at 10 rad/s and 0.5% strain. Statistical analyses were performed using a one‐way ANOVA. Data shown as mean ± SD of N = 3 hydrogels. P values indicating significance, ns >0.05, and ** ≤ 0.01.

Control glass‐bottom plates were coated with 10 µg/mL human fibronectin plasma for a minimum of 3 h at 37 °C. Human induced pluripotent stem cell‐derived cardiomyocytes iCell^2^ (CDI, Madison, USA) were thawed according to the manufacturer's instructions in iCell Plating Medium at RT. Cell suspension was centrifuged at 200 × g for 5 min, supernatant removed, and resuspended using the desired volume of iCell Maintenance Medium at 37 °C to achieve 750 000 cells mL^−1^. PBS++ (+Ca^2+^ +Mg^2+^) was removed from pre‐curated hydrogels and control glass‐bottom plates, and a 3 mm silicone stencil was placed on top of the hydrogel or coated area (control) (Figures 1A). A pre‐determined volume of cell suspension (30 µL) was carefully pipetted into each stencil to achieve 22 500 cells/stencil in a 3mm area. Media changes were performed every 48 h using iCell Maintenance Medium.

### 2.3 Spatial Analysis of Cell Movement

Cell movement within a 200 × 200 µm^2^ area was recorded on days 2 and 5 in vitro using a brightfield video using a high‐speed camera for 8 s (Hamamatsu ORCA‐flash 4.0 V2 digital CMOS camera running at 100 fps, 600 × 600 pixels, 200 × 200 µm^2^) and a 40× objective (Olympus, air objective). Cultures were maintained in incubated conditions at 37 °C, 5% CO_2_, and 80% humidity (CellOPTIQ, Clyde Biosciences, UK). Spatial analysis was used to study the contraction using a previously established algorithm, MUSCLEMOTION [32], implemented in software (ContractilityTool, Clyde Biosciences Ltd, UK) [33].

In this implementation, a grid of 10 × 10 squares (Figure S3) was created across the image, and several parameters were analyzed: amplitude of the contraction within each square; percentage of single peaks–a measure of the complexity of the contraction; and duration in milliseconds of 50% of the contraction (CD50).

### 2.4 Electrophysiology

The voltage‐sensitive dye approach was chosen over more conventional electrophysiological methods. The most commonly used approaches are based on an array of fixed surface electrodes to measure extracellular potential or injury potential, but both are unsuitable for this study because: (i) varying substrate stiffness while incorporating a fixed electrode array would be technically challenging, and (ii) extracellular potential signals from cardiomyocyte monolayers produce differential voltage signals (electrograms) that miss many important features of the transmembrane action potential signal. Injury potentials are not recommended as they are not stable over time. The voltage‐sensitive dye method has been validated in several studies as a viable approach for measuring electrical activity in iPSC‐derived cardiomyocytes and has the advantage of being non‐invasive and compatible with different substrate conditions.

Serum was removed from the medium by changing from iCell Maintenance Medium to a serum‐free solution (SFS): 120 mM NaCl, 20 mM HEPES, 5.40 mM KCl, 0.52 mM NaH_2_PO_4_, 3.5 mM MgCl_2_6H_2_O, 20 mM taurine, 20 mM creatine, and 11.1 mM glucose, with a pH of 7.4 at 37°C, on day 6 in vitro. Cell movement was recorded on day 7 due to cell viability on the gels, as previously described in the section above, in the absence of serum. Cultures were then loaded with 1:1000 FluoVolt and 1:100 PowerLoad (Life Technologies) in SFS for 25 min at 37 °C 5% CO_2_, and then the medium was changed to SFS. The cultures were settled for 30 min before electrophysiology was recorded. The voltage signal was recorded using a CellOPTIQ platform (Clyde Biosciences, Ltd), from a 200 × 200 um^2^ area using a 40× 0.6 numerical aperture (NA) objective lens. The excitation wavelength was 470 ± 10 nm using a light‐emitting diode (LED), and the emitted light was collected by a photomultiplier (PMT) at 590–650 nm. The fluorescent signal was digitized at 10 kHz, and the fluorescent signal was used to assess the time course of the transmembrane potential independent of cell movement [34, 35].

Cultures were maintained in an incubated stage (CellOPTIQ), and carbon electrodes were fitted. Electrophysiology and cell movement were recorded using CellOPTIQ or Contractility Tool, respectively, at spontaneous rates, 1, 2, and 3 Hz, then 1 Hz and returned to spontaneous. Analysis was performed in the same software as the recording.

### 2.5 Drug Study

Fibronectin‐PEG gels (5% 4‐arm‐PEG‐maleimide) were prepared in 96‐well glass‐bottom plates using 19.6 µL to achieve a 1 mm height in each well. The gels and control glass‐bottom plates were coated with 10 µg/mL human fibronectin plasma diluted in DPBS (+Ca^2+^ +Mg^2+^, DPBS++). iCell^2^ hiPSC‐CMs were plated at a density of 250 000 cells/mL, 25 000 cells/well. Thawing was done in iCell plating medium, and cells were further centrifuged at 1020 rpm for 5 min, and resuspended in iCell maintenance medium. On day 3 in vitro, the maintenance medium was replaced with SFS. On day 4, cell cultures were loaded with voltage‐sensitive dye 1:1000 FluoVolt and 1:100 PowerLoad (Life Technologies, UK) for 25 min, prepared in SFS, which was then replaced with SFS at 37 C 5% CO_2_. The cell culture was allowed to settle for 30 min before contractility and electrophysiology were recorded using a 40× objective. A red filter was used so that contractility using brightfield and electrophysiology could be recorded simultaneously.

The drugs selected included the β1‐adrenoceptor (β1‐AR) agonist isoproterenol; the LTCC blocker nifedipine; the antifungal itraconazole; and the selective cardiac myosin activator (myotrope) Omecamtiv mecarbil (OM). Drug powders were dissolved in DMSO as per the manufacturer's instructions to a final concentration of 0.1% DMSO [36]. During the drug addition to the cells, 50% of the well volume was replaced with the drug solution, reaching the target concentration in each well. The same procedure was done for vehicle control using DMSO. After 30 min incubation, contractility and electrophysiology were recorded as before, and cell cultures were paced at 1.2 Hz, 6ms 40 V pulses to overcome the intrinsic rate.

### 2.6 Biochemistry

Cell cultures were fixed for 10 min in 2% paraformaldehyde and washed 3 times for 3 min in Dulbecco's phosphate‐buffered saline without Ca^2+^ and Mg^2+^ (DPBS–). For immunofluorescent studies, cell cultures were fixed for 10 min in 2% paraformaldehyde (PFA) for 10 min and washed 3 times for 5 min with PBS—(without Ca^2+^ and Mg^2+^). Cultures were permeabilized at 4 °C for 5 min in 0.5% triton‐X diluted in PBS–. Block was done in 1% bovine‐serum albumin (BSA) for 30 min at room temperature. Primary antibodies (ab) were prepared at 1:200 ab:solution in 1% BSA, rhodamine phalloidin was added at 1:50 in 1% BSA, and incubated for 1 h at 37 °C. Cultures were washed three times with 0.5% PBST (0.5% tween‐20 prepared in PBS–), and secondary antibodies were added, prepared in 1% BSA at 1:200 ab:solution for 1h at 37 °C. Cultures were washed 3 times in PBST. Wells were emptied and drops of mounting medium with DAPI (VECTASHIELD antifade mounting media, Vector Labs) were added until the culture was fully covered, before a coverslip was laid on top. Samples were flipped so cells were the closest to the coverslip side. Images were taken with a ZEISS Axiobserver Z.1 with a 63x oil immersion objective. Immunofluorescent images were analyzed using a FIJI macro (see script in Appendix A).

For In‐Cell Western, the same antibodies were used. Cultures were fixed as before, and permeabilized with a perm buffer (301 mM sucrose, 50 mM NaCl, 630 µM MgCl_2_, 20 mM HEPES, 0.5% Triton‐X in PBS, pH 7.2), for 4 min at 4 °C. The culture was further washed with 1% milk protein diluted in PBS for 1.5 h. Primary antibodies prepared in 1% milk protein were incubated overnight at room temperature at 1:100 (except anti‐Cx43, which was 1:500). Cultures were washed five times with 0.1% Tween20 in PBS before the secondary. Secondaries were prepared at 1:800 in 1% milk protein, 0.2% tween 20, and 1:500 Li‐Cor Cell Tag 700 of the same species. Cultures were washed as before and allowed to dry overnight at 4 °C, before the plate reading at 800 nm for the protein of interest and 700 nm for the cell membrane. The data obtained was analyzed on Image Studio Lite 5.2. For further analysis, 700 nm fluorescence was divided by 800 nm fluorescence to correct for the amount of cell membrane. Plots and statistical analysis were performed on GraphPad Prism 9.

### 2.7 Rheology

To perform rheology measurements, hydrogels were prepared in 12 mm diameter PDMS molds using 250 µl volumes and left in PBS at 37 °C. Samples were subsequently measured with a Physica MCR 301 rheometer (Anton Parr) at 48 h post formation (Day 2). Samples were characterized at room temperature (∼23 °C) and sample hydration was ensured by pipetting PBS to the sides of the hydrogel whilst performing measurements. A frequency sweep from 100 to 1 rad/s was initially performed to determine the linear viscoelastic (LVE) regime of the hydrogels. Then, strain sweeps were performed at 10 rad/s in the range of 0.1% to 10%, within the LVE regime of the gels, and the shear modulus was determined at a gap size corresponding to a normal force of ≈0.1 N for all gels.

## 3 Results and Discussion

### 3.1 hiPSC‐CM Mechanics and Electrophysiology on PEG‐Fibronectin Hydrogels

In this study, we employed a methodology for culturing hiPSC‐CMs on FN‐PEG hydrogels, utilizing 6 mm PDMS molds for hydrogel preparation (Figure 1A). A silicone stencil was applied to the hydrogel surface, allowing for the precise insertion of hiPSC‐CMs. This approach facilitated the evaluation of cell behavior and helped hiPSC‐CMs to stay in close contact, contributing to our understanding of cellular interactions in engineered tissue models.

The mechanical properties of the extracellular matrix play a fundamental role in modulating hiPSC‐CMs behavior and maturation [37]. To establish which hydrogels are physiologically relevant substrates, rheological measurements were performed (see Figure 1B–D). Hydrogels were prepared at different wt. % using either 4‐arm or 8‐arm PEG hydrogels, and the shear modulus was obtained by performing a strain sweep after a frequency sweep was done (Figure 1B). The storage modulus (*G′*) of the 5 wt. % 4‐arm PEG hydrogels were found to be 4.3 kPa ± 0.5; the 5 wt. % 8‐arm PEG hydrogels were 2.2 kPa ± 0.4, and the 10 wt. % 4‐arm PEG hydrogels was 4.5 kPa ± 0.3, at 0.5% strain (Figure 1C). These results confirm that the hydrogels here developed display tunable stiffness ranging from 2–4 kPa, which is within the range of neonatal heart tissue (3.1–13.5 kPa) [11, 12].

Finally, there were no significant differences between the viscous moduli (tan *δ*) of the hydrogels (Figure 1D).

**FIGURE 2:**
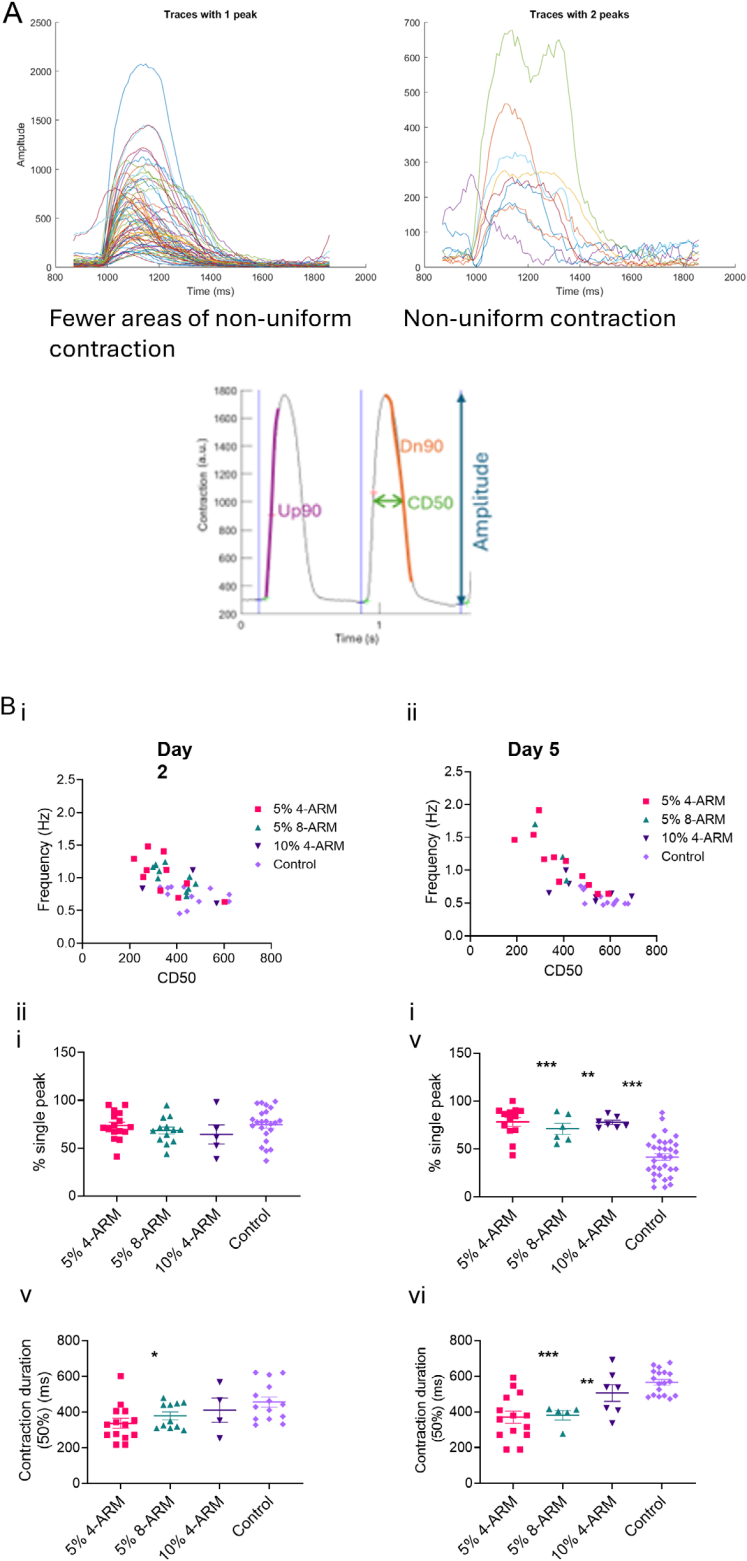
Correlation between thickness and mechanical properties of hiPSC‐CMs on hydrogels of different stiffnesses. Panel (A) shows an example record of spatial analysis done on a recording using CellOPTIQ. On the left are shown all traces from a single recording that exhibit a single peak, which represents a more uniform contraction, and to its right are the traces showing 2 or more peaks, representing a non‐uniform contraction of the tissue. The right figure shows how Time for contraction (Up90, purple), Time for relaxation (Dn90, orange), 50 % of contraction duration (CD50, green), and amplitude of contraction (blue) are measured using specialized software. (B) (i) frequency and CD50 correlation on day 2 in vitro; (ii) frequency and CD50 correlation on day 5 in vitro; (iii, iv) Contraction complexity on days 2 and 5 in vitro shown as the percentage of single peaks in a 200 × 200 um^2^ area using a 10 × 10 grid, respectively; (v, vi) duration of 50 % of the contraction on days 2 and 5 as a result of different stiffnesses. N = 3 platings, n >14 wells. One‐way ANOVA of hydrogel vs Control. *p*<0.001^***^, *p*<0.05^**^, *p*<0.01 ^*^.

The relationship between contraction duration (50%) (CD50) and beating frequency (Frequency) showed that as frequency increased, CD50 was also shorter, which means contraction is more stable (Figure 2B‐i,iii,v). This is opposed to controls where contraction duration starts to prolong over time. On day 5, the frequency increases more significantly in 5% 4‐arm (Figure 2B‐ii,iv,vi). The percentage of single peaks is representative of a uniform contraction in the tissue as displayed in Figure 2B‐iii. Day 2 shows this to be similar across all hydrogels and control (2D model on glass). On day 5, the percentage of single peaks in 5%‐4‐arm increases to 78.4 ± 4.5 %, in 5 % 8‐arm to 71.1 ± 5.9 %, and in 10 % 4‐arm to 77.8 ± 2.2 % from 41.6 ± 3.5 % in control, as shown in Figure 2B‐iv. Contraction duration increases overall from day 2–5, whereas 5 % 4‐arm exhibited a shorter CD50 at 371.0 ± 33.8 ms, as well as 5 % 8‐arm at 381.5 ± 46.6 ms compared to control (566.7 ± 16.3 ms) Figure 2B‐v,vi. This data shows that, at 5 days in vitro, hiPSC‐CMs cultured on 3D substrates show more uniform and simple contractions.

**FIGURE 3:**
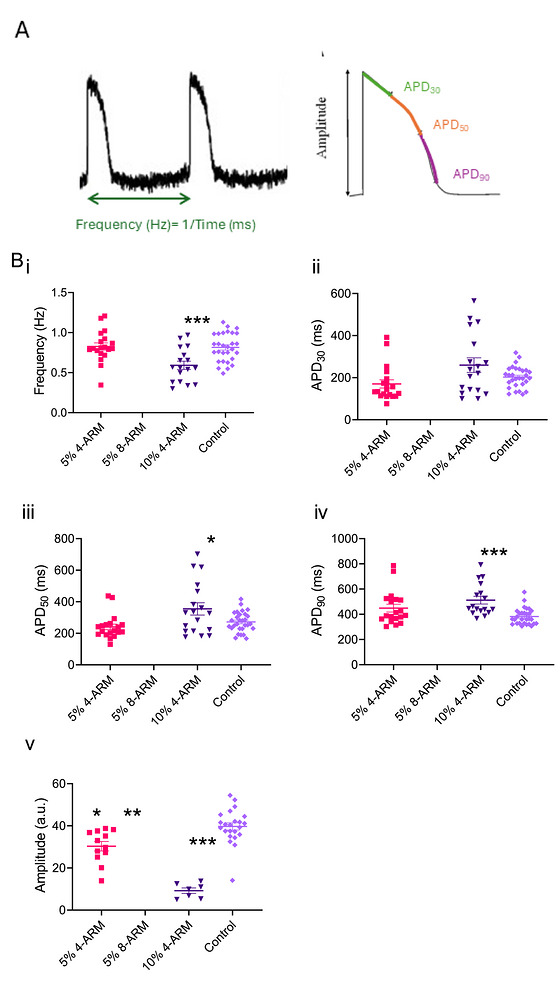
Electrophysiological behavior of hiPSC‐CMs cultured on different FN‐PEG gels at day 5 in vitro. Panel (A) shows an example trace of multiple action potentials. Frequency (Hz) of contraction is measured as 1/time between the start of each action potential. The duration of action potential is measured at 30 % or repolarization (APD30, green), 50 % (APD50, orange), and 90 % (APD90, purple). Panel (B) shows (i) spontaneous frequency; Action potential duration at 30 %, 50 %, and 90 % repolarization shown in 11), (iii), and iv), respectively. v) Amplitude of the voltage signal was also assessed for all hydrogels. N = 3 platings, n >14 wells. One‐way ANOVA of hydrogel vs Control. *p*<0.001^***^, *p*<0.05^**^, *p*<0.01 ^*^.

Electrophysiological parameters were measured using voltage‐sensitive dyes. The duration of action potentials was measured at 30 %, 50 % and 90 % to assess the different repolarization phases (Figure 2B‐ii–iv). Amplitude of the voltage signal was also assessed for all hydrogels (Figure 3B‐v).

It is worth mentioning that the 5 % 8‐ARM gel system exhibited substrate properties that adversely affected the hiPSC‐derived cardiomyocytes, preventing them from forming a functional monolayer. The cells failed to spread out and form a syncytial monolayer of spontaneously beating cells, and the voltage‐sensitive dye loading conditions were unable to restore normal cellular behavior.

The failure—characterized by a change in morphology to spherical shapes and subsequent detachment—was acute following a Voltage‐Sensitive Dye (VSD) introduction, suggesting a specific incompatibility between the extremely soft substrate (2.2 ± 0.4 kPa) and the robust spreading required for stable syncytial function under assay stress.

As such, no electrophysiological measurements were obtained from this condition.

Action potential duration remained unchanged across different hydrogels except for 10 % 4‐arm. This higher stiffness gel led to more variability in APD50 and 90 (Figure 3B‐iii,iv). The amplitude of the signal was smaller on all gels compared to the control (39.7 ± 1.7 a.u.), where 5 % 4‐arm was 30.3 ± 2.3 a.u. (Figure 3B‐v). More noticeably, the signal was much smaller at 9.2 ± 1.3 a.u. in 10 % 4‐arm. Due to the more uniform contractions seen on 5 % 4‐arm (Figure 2B) and unchanged electrophysiology (Figure 3B), this hydrogel was chosen for subsequent experiments, as this formulation was deemed most robust.

### 3.2 hiPSC‐CM Form Matrix and Cell–Cell Adhesive Complexes on PEG‐Fibronectin Hydrogels

**FIGURE 4:**
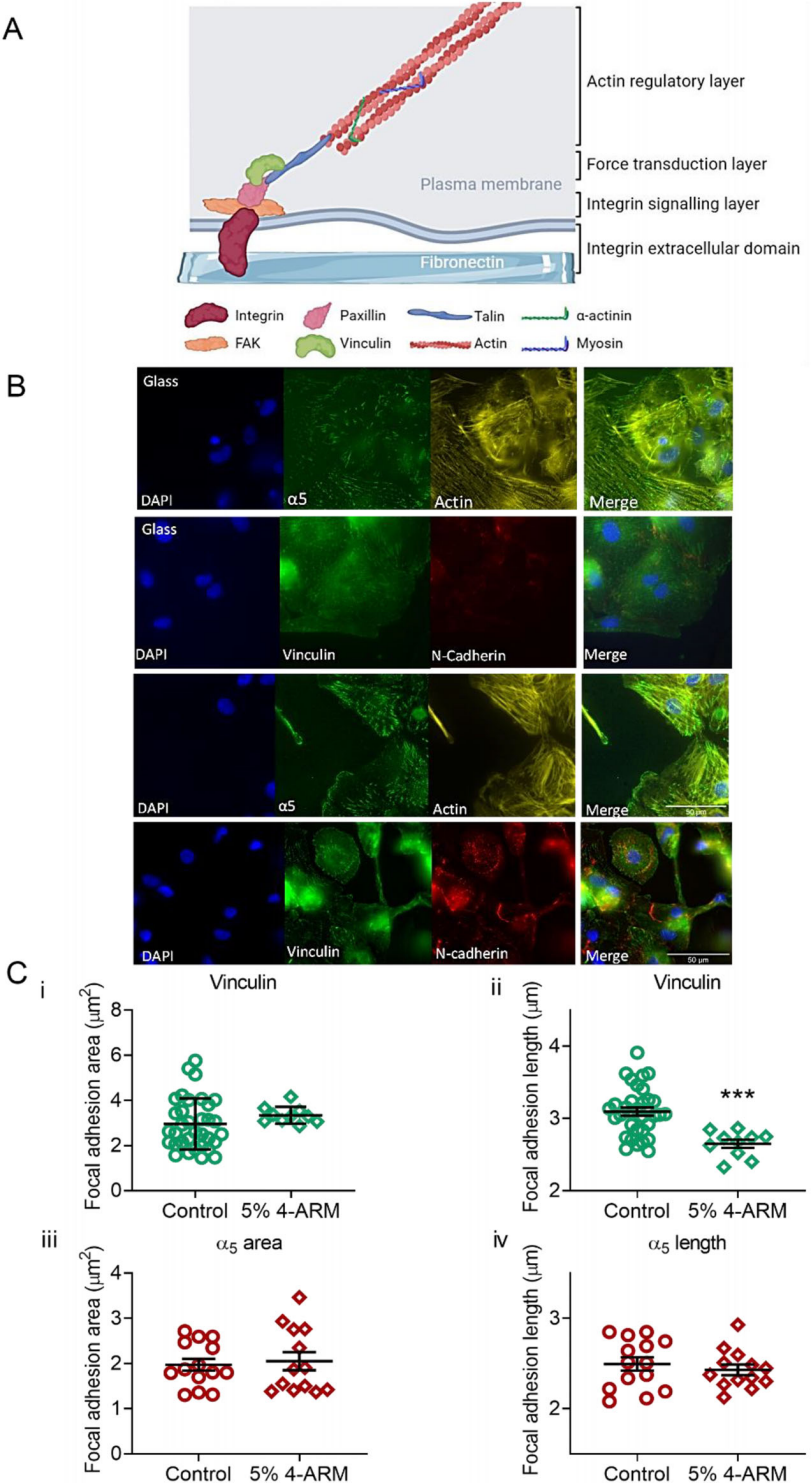
Cell‐to‐matrix adhesion between hiPSC‐CMs and 5 % 4‐arm FN‐PEG gel. (A) Diagram of protein interaction between cells and matrix (based on Salmerón‐Sánchez and Dalby, 2016) [39]; (B) Immunofluorescent images of hiPSC‐CMs on glass (control) and 5 % 4‐arm FN‐PEG gel showing nucleus (DAPI), vinculin adhesions, alpha‐5 integrin, n‐cadherin, and actin. The final image is merged. (C) (i) Vinculin area in cell; (ii) length of vinculin protein; (iii) area occupied by alpha‐5 protein; (iv) length of alpha‐5.

The effective use of hiPSC‐CMs in cardiac regenerative medicine and drug screening relies heavily on their capacity to form functional interactions with the substrate they are grown on, mimicking the native cardiac microenvironment [38]. Figure 4A provides a schematic of the key proteins that mediate cell‐matrix interaction [39]. Immunofluorescence images of hiPSC‐CMs cultured on 5 % 4‐arm FN‐PEG hydrogels revealed the formation of well‐developed cell‐matrix adhesions (Figure 4B). Vinculin, a focal adhesion protein, showed distinct localization at the cell‐substrate interface, indicating robust mechanical coupling between cells and the hydrogel matrix [40]. Immunofluorescence for α5 integrin, a primary fibronectin receptor, demonstrates specific engagement with the fibronectin‐functionalized hydrogel surface. Additionally, the expression of N‐cadherin at cell‐cell interfaces indicates the development of intercellular connections necessary for synchronized cardiac function [41].

Quantitative analysis of adhesion complex morphology provided deeper insights into the cell‐matrix interactions (Figure 4C). Vinculin area measurements (Figure 4C‐i) indicated the formation of substantial focal adhesions, suggesting appropriate substrate engagement comparable with the glass control. The vinculin length distribution (Figure 4C‐ii) revealed mature focal adhesions, though at lower levels than the glass control. The α5 integrin distribution analysis, examining both area (Figure 4C‐iii) and length (Figure 4C‐iv), indicated effective fibronectin‐mediated cell adhesion, confirming overall cell‐matrix interactions comparable to those of glass controls.

The presence of well‐developed adhesion complexes, coupled with specific integrin engagement and cytoskeletal organization, suggests that these substrates can support proper hiPSC‐CM maturation and contractile function [19].

For hiPSC‐CMs to serve as effective models for cardiac tissue or as therapeutic agents, they need to establish robust cell–cell adhesions that enable synchronized contractions and electrical signal propagation [41].

**FIGURE 5:**
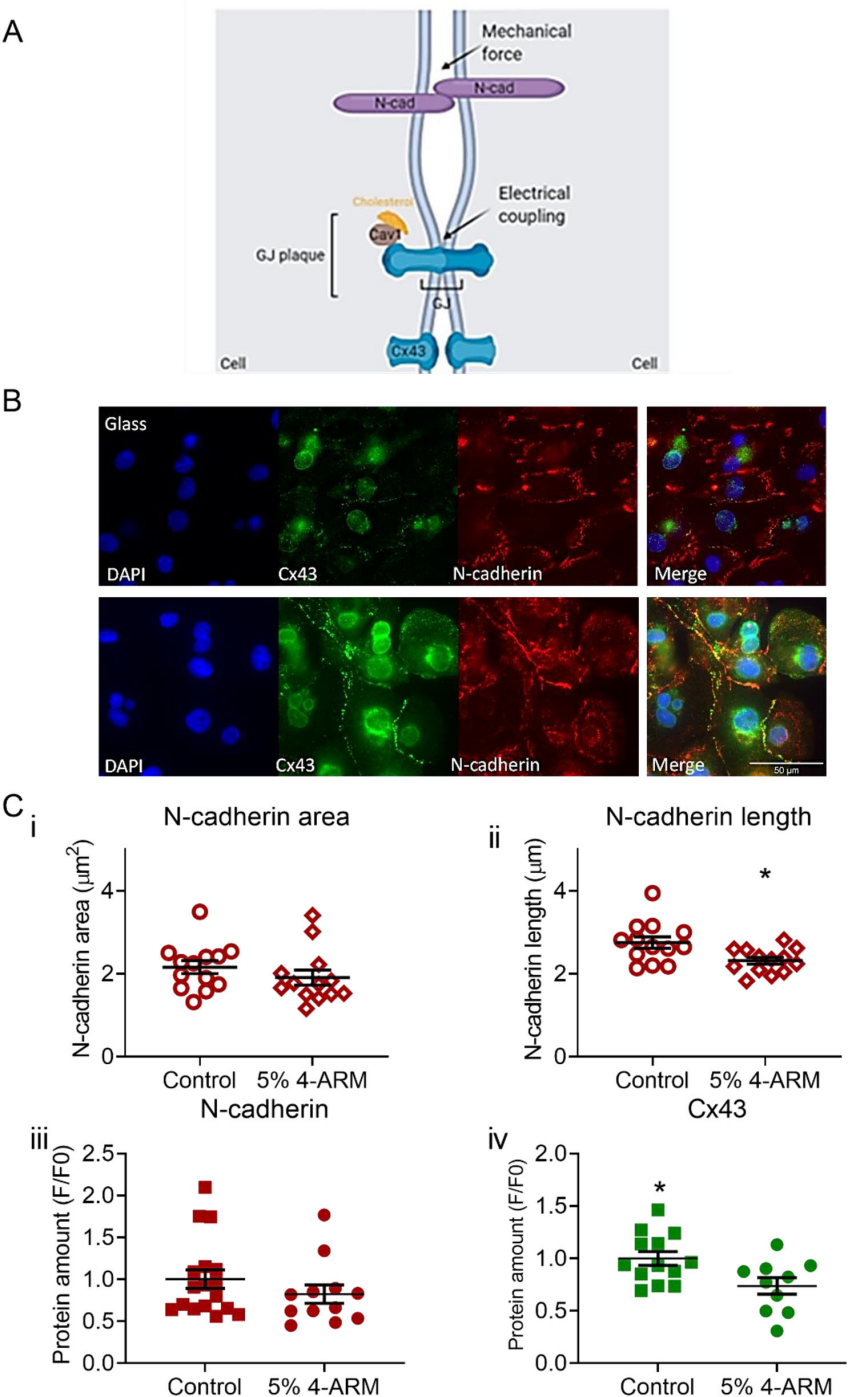
Cell‐to‐cell adhesion between hiPSC‐CMs on 5 % 4‐arm FN‐PEG gel. (A) Diagram of protein interaction between cells (based on [44]). (B) Immunofluorescent images of hiPSC‐CMs on glass (control) and 5 % 4‐arm FN‐PEG gel showing nucleus (DAPI), C×43, and n‐cadherin. The final image is merged. (C) (i) N‐cadherin area in cell; (ii) length of N‐cadherin protein; (iii) protein quantification of N‐cadherin using InCell Western; iv) C×43 quantification. All data in panels (iii, iv) were normalized by calculating F/F0 (Protein fluorescence/control fluorescence, where control was composed of wells without primary antibody.

Figure 5A illustrates the key molecular components involved in cell–cell communication and mechanical coupling between adjacent cardiomyocytes.

Immunofluorescence images revealed the formation of N‐cadherins and gap junctions on the hydrogel substrate (Figure 5B). N‐cadherin, an essential component of adherens junctions, showed distinct localization at cell–cell interfaces, indicating the development of mechanical junctions necessary for force transmission between adjacent cells. Notably, the presence of connexin 43 (C×43) at cellular interfaces demonstrates the establishment of gap junctions, which are crucial for electrical coupling and ion transfer between cells [42]. Quantitative analysis of N‐cadherin expression showed substantial adherens junction formation per cell (Figure 5C‐i). Measurements of N‐cadherin protein length distributions (Figure 5C‐ii) revealed mature adherent junctions, though smaller than those observed in the glass control. In‐cell Western analysis of N‐cadherin protein levels demonstrated robust expression of this crucial adhesion molecule (Figure 5C‐iii). Similarly, quantification of C×43 protein levels, normalized to cell membrane expression and presented on a scale of 0–1, indicated abundant gap junction formation (Figure 5C‐iv), supporting the establishment of electrical coupling between adjacent cells.

These quantitative measurements provide strong evidence that hiPSC‐CMs cultured on 5 % 4‐arm FN‐PEG hydrogels develop both mechanical and electrical intercellular connections. The simultaneous presence of robust adherens junctions and gap junctions suggests the formation of functional syncytial networks capable of coordinated contractile activity [43]. These characteristics are essential for applications in tissue engineering and drug screening, where synchronized cellular behavior and force transmission are critical requirements.

### 3.3 hiPSC‐CM Response to Pharmacological Agents on PEG‐Fibronectin Hydrogels

We then sought to investigate the response of hiPSC‐CM to different known cardioactive compounds in order to assess the drug‐screening suitability of the presented 3D hiPSC‐CM‐PEG‐fibronectin platform (5 % 4‐arm FN‐PEG gel) compared to conventional 2D glass controls.

**FIGURE 6:**
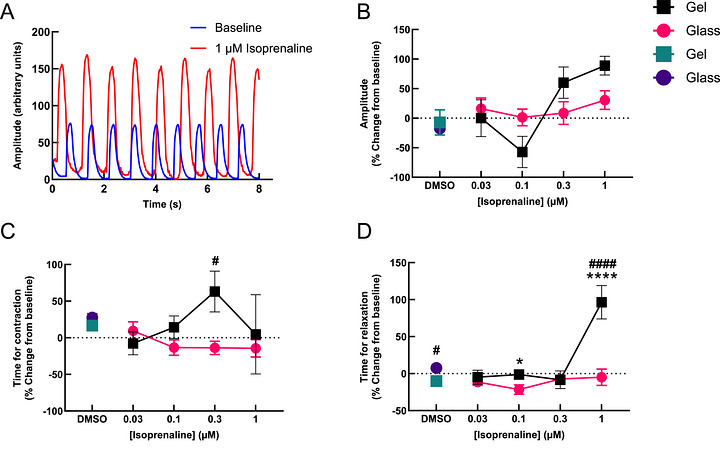
Isoprenaline effects on cardiomyocyte spontaneous movements. A contraction amplitude trace showing the inotropic effect of 1 µM isoprenaline when hiPSC‐CMs were cultured on gel in a specific cell culture well (A) was produced by the ContractilityTool. Three main parameters were used to investigate isoprenaline effects on hiPSC‐CMs cultured in 2D or on hydrogels: contraction amplitude (B), contraction time (C), and relaxation time (D). For every parameter mean ± SEM was plotted, n ≧2 wells. Asterisk symbols (*) mark a significant difference between different drug concentrations and control (DMSO) treatments (blue * for glass, orange * for gel). The number sign symbols (#) mark a significant difference between gel and glass conditions. One and two‐way ANOVA analysis was performed: ^*^/# ‐ *p*‐value≤ 0.05; ^**^/## ‐ *p*‐value≤ 0.01; ^***^/### ‐ *p*‐value≤ 0.001; ^****^/#### ‐ *p*‐value≤ 0.0001.

First, we tested isoprenaline, a β‐adrenergic receptor agonist that is known to induce positive inotropic effects, such as increased contractility through activation of L‐type calcium channels [45]. Four concentrations of isoprenaline ranging from 0.03 to 1 µM were studied in hiPSC‐CMs cultured on 5wt. % 4‐armhydrogels compared to a glass control (Figure 6). Amplitude, time for contraction, and time for relaxation were measured at different concentrations of the drug. These are parameters of the contraction that indicate the time for the tissue to contract spontaneously, to relax, and the size of the contraction (amplitude). Contraction amplitude did not change in response to isoprenaline treatment (Figure 6B), yet a positive trend was noted at the highest studied concentration, 1 µM (Figure 6A). No change in contraction time was detected in response to isoprenaline treatment (Figure 6C), however, data showed that at 0.3 µM isoprenaline treatment, hiPSC‐CMs cultured on gel (+63.1 ± 27.8 %) and glass (−13.8 ± 9.1 %) exhibited significantly different responses. Cardiomyocytes cultured on glass treated with 0.1 µM (−21.6 ± 6.4 %) showed a decrease of 29 % in time to reach 90 % of relaxation (Dn90) compared to DMSO (+7.5 ± 3.6 %) (Figure 6D). In 3D cell culture, 1 µM isoprenaline treatment (+96.4 ± 22.5 %) led to an increase of 107 % in comparison to DMSO (−10.1 ± 4.5 %).

**FIGURE 7:**
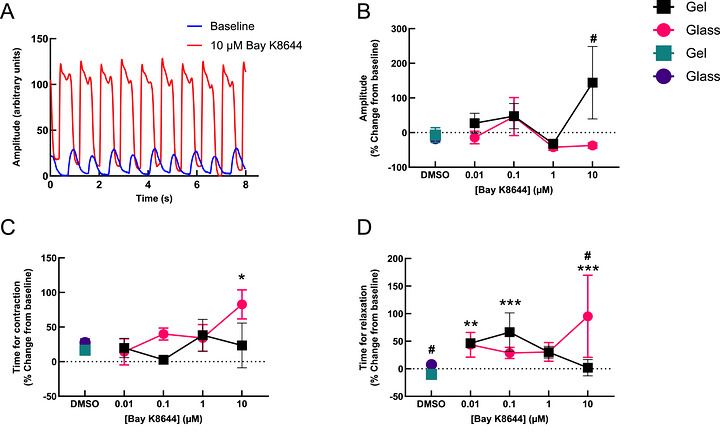
BayK8644 effects on cardiomyocyte spontaneous movements. A contraction amplitude trace showing the inotropic effect of 10 µM BayK8644 when hiPSC‐CMs were cultured on gel in a specific cell culture well (A) was produced by the ContractilityTool. Three main parameters were used to investigate BayK8644 effects on hiPSC‐CMs cultured in 2D or 3D environments: contraction amplitude (B), contraction time (C), and relaxation time (D). For every parameter mean ± SEM was plotted, n≧4 wells. Asterisk symbols (*) mark a significant difference between different drug concentrations and control (DMSO) treatments (blue * for glass, orange * for gel). The number sign symbols (#) mark a significant difference between gel and glass conditions. One and two‐way ANOVA analysis was performed: ^*^/# ‐ *p*‐value≤ 0.05; ^**^/## ‐ *p*‐value≤ 0.01; ^***^/### ‐ *p*‐value≤ 0.001; ^****^/#### ‐ *p*‐value≤ 0.0001.

Next, we tested BayK8644, a direct L‐type calcium channel agonist which also has positive inotropic effects [46]. In the present study, a trend toward a positive inotropic effect of BayK8644 was seen in both gel and glass‐cultured hiPSC‐CMs. The effects of 4 concentrations of BayK8644, ranging from 0.01 to 10 µM, were studied in hiPSC‐CMs cultured on 5wt. % 4‐ARM hydrogels compared to a glass control. No significant change in contraction amplitude was observed in response to BayK8644 treatment (Figure 7B), yet a positive trend was noted at the highest tested concentration, 10 µM (Figure 7A). BayK8644 effects on cells were seen to be significantly different between 2D (−37.3 ± 7.4 %) and 3D (+144 ± 104.5 %) cultures at 10 µM treatment (Figure 7B). Up90 increased with 10 µM BayK8644 (+82.8 ± 21 %) in glass culture (+27.5 ± 5.4 %) (Figure 7C). Increased hiPSC‐CM relaxation time in response to BayK8644 treatment was observed in both gel and glass conditions (Figure 7D). The time required for cardiomyocytes to reach the Dn90 stage was 54 % and 77 % greater in cells cultured on gel and treated with 0.01 µM (+46.3 ± 8.1 %) and 0.1 µM (+66.4 ± 34.9 %) BayK8644, respectively, in comparison to DMSO (−10.1 ± 4.5 %). Time to reach Dn90 was increased by 88 % in 2D cardiomyocytes treated with 10 µM (+95.2 ± 74.5 %) BayK8644 as compared to DMSO (+7.5 ± 3.6 %).

**FIGURE 8:**
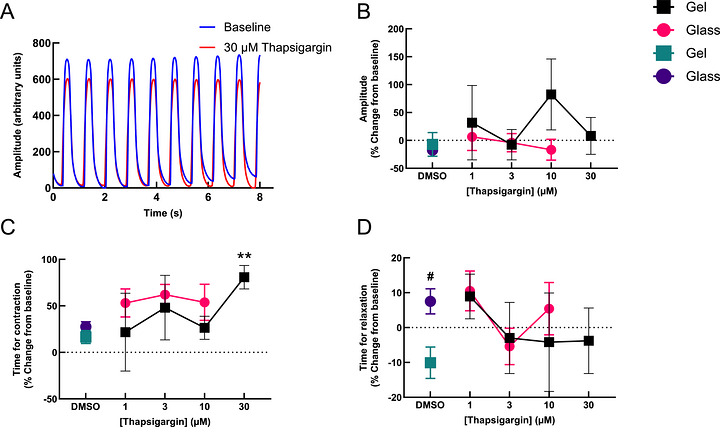
Thapsigargin effects on cardiomyocyte spontaneous movements. A contraction amplitude trace showing the inotropic effect of 30 µM thapsigargin when hiPSC‐CMs were cultured on gel in a specific cell culture well (A) was produced by the ContractilityTool. Three main parameters were used to investigate thapsigargin effects on hiPSC‐CMs cultured in 2D or 3D environments: contraction amplitude (B), contraction time (C), and relaxation time (D). For every parameter mean ± SEM was plotted, n≧3 wells. Asterisk symbols (*) mark a significant difference between different drug concentrations and control (DMSO) treatments (blue * for glass, orange * for gel). The number sign symbols (#) mark a significant difference between gel and glass conditions. One and two‐way ANOVA analysis was performed: ^*^/# ‐ *p*‐value≤ 0.05; ^**^/## ‐ *p*‐value≤ 0.01; ^***^/### ‐ *p*‐value≤ 0.001; ^****^/#### ‐ *p*‐value≤ 0.0001.

In addition, we tested thapsigargin, which has a known negative inotropic effect through the inhibition of sarcoendoplasmic reticulum calcium transport ATPase (SERCA), a calcium pump responsible for Ca^2+^ transport from the cytoplasm into the sarcoplasmic reticulum [47]. Four concentrations of thapsigargin ranging from 0.3 to 10 µM were studied in hiPSC‐CMs cultured on 5wt. % 4‐arm hydrogels compared to a glass control. Thapsigargin treatment did not affect the contraction amplitude of gel or glass conditions (Figure 8A,B). Up90 was prolonged in gel culture at 30 µM thapsigargin (+80.8 ± 12.6 %) by 64 % in comparison with DMSO (+16.5 ± 6.9 %) (Figure 8C). Thapsigargin did not affect hiPSC‐CM relaxation time (Figure 8D). No results were obtained from 2D hiPSC‐CM culture treated with 30 µM thapsigargin due to cell quiescence.

**FIGURE 9:**
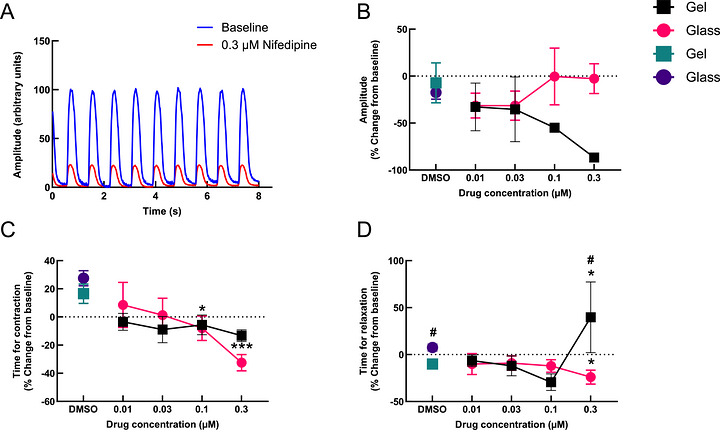
Nifedipine effects on cardiomyocyte spontaneous movements. A contraction amplitude trace showing the inotropic effect of 0.3 µM nifedipine when hiPSC‐CMs were cultured on gel in a specific cell culture well (A) was produced by the ContractilityTool. Three main parameters were used to investigate nifedipine effects on hiPSC‐CMs cultured in 2D or 3D environments: contraction amplitude (B), contraction time (C), and relaxation time (D). For every parameter mean ± SEM was plotted, n ≧2 wells. Asterisk symbols (*) mark a significant difference between different drug concentrations and control (DMSO) treatments (blue * for glass, orange * for gel). The number sign symbols (#) mark a significant difference between gel and glass conditions. One and two‐way ANOVA analysis was performed: ^*^/# ‐ *p*‐value≤ 0.05; ^**^/## ‐ *p*‐value≤ 0.01; ^***^/### ‐ *p*‐value≤ 0.001; ^****^/#### ‐ *p*‐value≤ 0.0001.

Finally, we tested the effects of nifedipine, an L‐type calcium channel inhibitor that exerts a negative inotropic effect by blocking calcium entry into cardiomyocytes and preventing their depolarization [45]. Again, four concentrations of nifedipine ranging from 0.01 to 0.3 µM were studied in hiPSC‐CMs cultured on 5wt. % 4‐arm hydrogels compared to a glass control. Nifedipine did not affect contraction amplitude (Figure 9B), yet a negative trend was observed at the highest nifedipine concentration on gel (Figure 9A). Contraction time decreased in glass culture by 36% and 60% at 0.1 µM (−8 ± 8.7%) and 0.3 µM (−32.5 ± 5.8%) nifedipine, respectively, in comparison to DMSO (+27.5 ± 5.4%) (Figure 9C). Cardiomyocytes exhibited a decrease in relaxation time of 32% on glass (−24 ± 7.4%) and an increase of 50% on gel (+39.7 ± 37.6%) at 0.3 µM nifedipine in comparison to glass (+7.5 ± 3.6%) and gel (−10.1 ± 4.5%) controls, respectively (Figure 9D).

## 4 Discussion

This study demonstrates the optimization of hydrogel substrates for culturing iPSC‐derived cardiomyocytes (iPSC‐CMs), yielding significant improvements in cardiomyocyte physiology. We observed that hydrogel culture significantly alters cell mechanics and electrophysiology, promoting more physiologically relevant properties compared to traditional 2D culture on glass (Figures 2 and 3). Furthermore, the enhanced formation of cell–cell and cell‐matrix adhesions within the hydrogel environment suggests improved structural integrity and functional connectivity (Figures 4 and 5).

These structural improvements are supported by rigorous, objective measurement modalities, including automated image analysis utilizing a validated FIJI macro for standardized quantification of Vinculin and N‐cadherin morphology, and In‐Cell Western (ICW) quantitative immunoassay. The ICW analysis specifically provided high‐throughput, bulk quantification of critical gap junction (Connexin 43) and adherens junction (N‐cadherin) protein levels, confirming robust expression essential for electrical coupling and intercellular force transmission.

The novelty of the present study lies in the synthetic, modular, and chemically defined nature of the FN‐PEG hydrogel platform. This PEG‐based chemistry allows for the orthogonal control of substrate stiffness—tuned precisely to the neonatal heart range (2–4 kPa)—whilst using fibronectin specifically to facilitate cell‐matrix adhesion via the integrin pathway. This approach enables systematic study and isolation of mechanical inputs in a scalable 2D format designed for drug toxicity screening. Importantly, our results demonstrate that drug responses are mechanosensitive, with differential kinetic changes observed between physiologically compliant hydrogels and rigid glass substrates. This improved environment may contribute to the observed positive inotropic effect of isoprenaline (Figure 6), which is a β‐adrenergic receptor agonist known to exhibit a positive inotropic effect. The inotropic effect seen in our data is also in agreement with previously published studies [48, 49, 50]. Additionally, contraction amplitude was seen to be increased in cardiomyocytes cultured both on gel and glass, showing a potential increase in contractile strength. An increase was also observed in relaxation time in hiPSC‐CMs cultured on hydrogel when treated with 1 µM isoprenaline. This finding was surprising as isoprenaline is normally known to enhance relaxation rate [51]. This contradictory result may be due to immaturity of hiPSC‐CMs as they lack a fully developed sarcoplasmic reticulum (SR) [52].

A study by Layland and Kentish [51] supported this result as it showed that inhibition of SR in rat cardiac tissues results in elimination of isoprenaline positive lusotropic effects, while the positive inotropy remained, as seen in the present study findings.

BayK8644 is a direct L‐type calcium channel agonist with a known positive inotropic effect [46]. In the present study, a trend toward a positive inotropic effect of BayK8644 was seen in both hydrogel and glass‐cultured hiPSC‐CMs (Figure 7). In gel conditions, BayK8644 triggered the highest contraction amplitude increase at 10 µM, and this effect was significantly different from glass‐cultured hiPSC‐CMs; however, the overall increase in contraction amplitude was not significant. In glass conditions, the positive inotropy was indicated by increased contraction time at 10 µM BayK8644. Such observations agreed with a previously published study [53] in which contraction amplitude and time were reported to be increased in response to BayK8644 treatment due to increased influx of Ca^2+^ ions. Furthermore, in the current study, BayK8644 was also shown to prolong hiPSC‐CM relaxation time and trigger disturbed contraction as seen from the trace in Figure 7A. These findings may be explained by previously reported data in the study of Sicouri et ;al. [54], where BayK8644 was shown to trigger ventricular extrasystoles and tachycardia in cardiomyocytes.

Thapsigargin is a negative inotrope that exerts its effects through inhibition of sarcoendoplasmic reticulum calcium transport ATPase (SERCA), which is a calcium pump responsible for Ca^2+^ transport from the cytoplasm into the sarcoplasmic reticulum [47]. In the presented research project, no definitive inotropic effect was observed on hiPSC‐CM contraction in the presence of Thapsigargin. Contrary to what was expected, thapsigargin was observed to increase contraction time in hydrogel cardiomyocyte culture. This finding was also reported in Hortigon–Vinagre et al. [48], where thapsigargin was shown to increase the time to cardiomyocyte depolarization. The mechanistic basis for this effect is uncertain, but this may be the basis for the increased time to contract and, therefore, contraction time.

Nifedipine is an L‐type calcium channel inhibitor that exerts a negative inotropic effect by blocking calcium entry into cardiomyocytes and reducing the contribution of the current to the plateau phase of the AP and subsequently causing reduced action potential duration [31].

In the current study, a trend toward negative inotropy was observed in contraction amplitude in hiPSC‐CMs cultured on gel in the presence of Nifedipine (Figure 9). Additionally, contraction time was shown to be significantly decreased in 2D hiPSC‐CM culture in response to nifedipine. These findings were in agreement with a study by Hortigon–Vinagre et al. [48], where nifedipine was reported to cause a concentration‐dependent contraction amplitude decrease in hiPSC‐CMs.

Additionally, in accordance with data shown in this work, a decrease in contraction amplitude in response to nifedipine treatment in hiPSC‐CMs and isolated guinea pig hearts was also reported in Ref. [55].

Overall, drug testing revealed distinct differences in drug responses between iPSC‐CMs cultured on hydrogels (3D) and glass (2D), indicating that hydrogel substrates can provide a more representative in vitro model for cardiotoxicity screening and drug discovery.

These findings are particularly significant given that previous studies have shown that the use of standard glass/plastic (inflexible) substrates for monolayer studies causes heterogeneous and abnormal contraction/relaxation events, which our results with flexible hydrogel substrates help to address [56].

The advantages of the cell monolayer configuration employed in this study are evident in our results: (i) the industry standard format ensured adequate oxygenation of the cell layer under standardised conditions, contributing to consistent drug responses; (ii) the monolayer structure allowed cell structures to be visible using normal microscope optics and served as reference points for contraction measurements; (iii) the confluent monolayer ensured that all cells were electrically coupled and functioned as an electrical syncytium, enabling coordinated responses to pharmacological interventions; (iv) the access of the cells to drugs in an aqueous serum free medium ensured the cells were exposed to a known free concentration of the drug, providing reliable dose‐response relationships that would be uncertain in a 3D aqueous gel structure given the various chemical partition coefficients of organic molecules.

Whilst we recognise that longer culture periods (beyond one week) are generally preferred for achieving advanced hiPSC‐CM maturation necessary for complex disease modelling, our study was technically constrained to a 7‐day culture due to limitations inherent to the commercially sourced hiPSC‐CMs within this novel synthetic matrix. Crucially, this 7‐day period was sufficient to establish the primary conclusions of the work: defining the role of physiological stiffness (2–4 kPa) in normalizing contractile behavior (improved uniformity) and demonstrating that drug responses are mechanosensitive, as evidenced by the profound differential kinetic changes observed with isoprenaline and nifedipine compared to the rigid control. Thus, the study achieved its objective of validating the FN‐PEG platform as a superior model for early‐stage, predictive cardiotoxicity screening. While our results demonstrate that hydrogel substrates provide more physiologically relevant mechanical properties compared to traditional glass substrates, it is important to acknowledge that these alternative substrates may introduce additional chemical and mechanical interactions that could influence drug responses. Some of these processes may be physiological, while others may be unique to the gel format used. In both cases, these interactions and their modulation by drugs add an additional layer of complexity to the mechanical and electrical response of cardiomyocytes, which requires further investigation and consideration in drug discovery and screening applications. These findings highlight the potential of optimized hydrogels to create more predictive and relevant platforms for cardiac research and drug development.

## Author Contributions

A.D.S.C., G.S., and M.S.‐S. conceived the project. A.D.S.C. designed, performed, and analyzed most of the experiments. L.S. performed some analysis on the drug toxicity study and wrote the respective section of the discussion. S.T. and O.D. designed the hydrogels. M.A.G.O. performed the mechanical analysis of the hydrogels. F.B. is responsible for creating the data analysis software: CellOPTIQ and Contractility Tool, and helped develop the macro for IHC analysis. A.D.S.C., O.D., and L.S. wrote the manuscript that was edited by M.A.G.O., G.S., and M.S.S. All authors read the manuscript. O.D., M.J.D., G.S., and M.S.S. supervised the project and acquired funding.

## Conflicts of Interest

The authors declare no conflict of interest.

## Supporting information




**Supporting file**: adhm70519‐sup‐0001‐SuppMat.docx

## Data Availability

The data that support the findings of this study are available from the corresponding author upon reasonable request.

## Appendix A

### A.1

**Macro FIJI for Immunofluorescence Analysis**:

## References

[1] N. Silbernagel , A. Körner , J. Balitzki , et al., “Shaping the Heart: Structural and Functional Maturation of iPSC‐Cardiomyocytes in 3D‐Micro‐Scaffolds,” Biomaterials 227 (2020): 119551, 10.1016/j.biomaterials.2019.119551. 31670034

[2] S. Kussauer , R. David , and H. Lemcke , “hiPSCs Derived Cardiac Cells for Drug and Toxicity Screening and Disease Modeling: What Micro‐ Electrode‐Array Analyses Can Tell Us,” Cells 8 (2019): 1331, 10.3390/cells8111331. 31661896 PMC6912416

[3] M. Csöbönyeiová , Š. Polák , and L. Danišovič , “Toxicity Testing and Drug Screening Using iPSC‐Derived Hepatocytes,” Journal of Physiology and Pharmacology 94, no. 7 (2016): 687–694, 10.1139/cjpp-2015-0459. 27128322

[4] K. Takahashi and S. Yamanaka , “Induction of Pluripotent Stem Cells From Mouse Embryonic and Adult Fibroblast Cultures by Defined Factors,” Cell 126, no. 4 (2006): 663–676, 10.1016/j.cell.2006.07.024. 16904174

[5] N. Stockbridge , J. Morganroth , R. R. Shah , and C. Garnett , “Dealing With Global Safety Issues,” Drug Safety 36, no. 3 (2013): 167–182, 10.1007/s40264-013-0016-z. 23417505

[6] K. Blinova , J. Stohlman , J. Vicente , et al., “Comprehensive Translational Assessment of Human‐Induced Pluripotent Stem Cell Derived Cardiomyocytes for Evaluating Drug‐Induced Arrhythmias,” Toxicological Sciences 155, no. 1 (2017): 234–247, 10.1093/toxsci/kfw200. 27701120 PMC6093617

[7] B. Fermini and A. A. Fossa , “The Impact of Drug‐Induced QT Interval Prolongation on Drug Discovery and Development,” Nature Reviews Drug Discovery 2, no. 6 (2003): 439–447, 10.1038/nrd1108. 12776219

[8] E. Dick , D. Rajamohan , J. Ronksley , and C. Denning , “Evaluating the Utility of Cardiomyocytes From Human Pluripotent Stem Cells for Drug Screening,” Biochemical Society Transactions 38, no. 4 (2010): 1037–1045, 10.1042/BST0381037. 20659000

[9] J. T. Koivisto , C. Gering , J. Karvinen , et al., “Mechanically Biomimetic Gelatin–Gellan Gum Hydrogels for 3D Culture of Beating Human Cardiomyocytes,” ACS Applied Materials & Interfaces 11, no. 23 (2019): 20589–20602, 10.1021/acsami.8b22343. 31120238 PMC6750838

[10] R. Lanza , Essentials in Stem Cell Biology, (Academic Press, 2006).

[11] M. Wheelwright , Z. Win , J. L. Mikkila , K. Y. Amen , P. W. Alford , and J. M. Metzger , “Investigation of Human iPSC‐Derived Cardiac Myocyte Functional Maturation by Single Cell Traction Force Microscopy,” PLoS ONE 13, no. 4 (2018): 0194909, 10.1371/journal.pone.0194909. PMC5884520 29617427

[12] F. B. Bedada , S. S. Chan , and L. Zhang , “Acquisition of a Quantitative, Stoichiometrically Conserved Ratiometric Marker of Maturation Status in Stem Cell‐Derived Cardiac Myocytes,” Stem Cell Reports 3, no. 4 (2014): 594–605, 10.1016/j.stemcr.2014.07.012. 25358788 PMC4223713

[13] P. W. Burridge , E. Matsa , P. Shukla , et al., “Chemically Defined Generation of Human Cardiomyocytes,” Nature Methods 11, no. 8 (2014): 855–860, 10.1038/nmeth.2999. 24930130 PMC4169698

[14] T. J. Herron , A. M. D. Rocha , K. F. Campbell , et al., “Extracellular Matrix–Mediated Maturation of Human Pluripotent Stem Cell–Derived Cardiac Monolayer Structure and Electrophysiological Function,” Circ: Arrhythmia and Electrophysiology 9 (2016): 003638, 10.1161/CIRCEP.113.003638. PMC4833010 27069088

[15] N. Hersch , B. Wolters , G. Dreissen , et al., “The Constant Beat: Cardiomyocytes Adapt Their Forces by Equal Contraction upon Environmental Stiffening,” Biology Open 2, no. 3 (2013): 351–361, 10.1242/bio.20133830. 23519595 PMC3603417

[16] A. J. Engler , C. Carag‐Krieger , C. P. Johnson , et al., “Embryonic Cardiomyocytes Beat Best on a Matrix With Heart‐Like Elasticity: Scar‐Like Rigidity Inhibits Beating,” Journal of Cell Science 121, no. 22 (2008): 3794–3802, 10.1242/jcs.029678. 18957515 PMC2740334

[17] M. F. Tenreiro , A. F. Louro , P. M. Alves , and M. Serra , “Next Generation of Heart Regenerative Therapies: Progress and Promise of Cardiac Tissue Engineering,” Regenerative Medicine 6, no. 1 (2021): 30, 10.1038/s41536-021-00140-4. 34075050 PMC8169890

[18] X. Wang , S. Yu , L. Xie , M. Xiang , and H. Ma , “The Role of the Extracellular Matrix in Cardiac Regeneration,” Heliyon 11, no. 1 (2025): 41157, 10.1016/j.heliyon.2024.e41157. PMC11745795 39834404

[19] Y. Guo and W. T. Pu , “Cardiomyocyte Maturation,” Circulation Research 126, no. 8 (2020): 1086–1106, 10.1161/CIRCRESAHA.119.315862. 32271675 PMC7199445

[20] P. Lu , D. Ruan , M. Huang , et al., “Harnessing the Potential of Hydrogels for Advanced Therapeutic Applications: Current Achievements and Future Directions,” Signal Transduction and Targeted Therapy 9, no. 1 (2024): 166, 10.1038/s41392-024-01852-x. 38945949 PMC11214942

[21] Q. Xu , Z. Xiao , Q. Yang , et al., “Hydrogel‐Based Cardiac Repair and Regeneration Function in the Treatment of Myocardial Infarction,” Materials Today Bio 25 (2024): 100978, 10.1016/j.mtbio.2024.100978. PMC10907859 38434571

[22] H. Ren , Y. Cheng , G. Wen , J. Wang , and M. Zhou , “Emerging Optogenetics Technologies in Biomedical Applications,” Smart Medicine 2, no. 4 (2023): 20230026, 10.1002/SMMD.20230026. PMC11235740 39188295

[23] Z.‐S. Razavi , M. Soltani , G. Mahmoudvand , et al., “Advancements in Tissue Engineering for Cardiovascular Health: A Biomedical Engineering Perspective,” Frontiers in Bioengineering and Biotechnology 12 (2024): 1385124, 10.3389/fbioe.2024.1385124. 38882638 PMC11176440

[24] S. S. Nunes , J. W. Miklas , J. Liu , et al., “Biowire: A Platform for Maturation of Human Pluripotent Stem Cell–Derived Cardiomyocytes,” Nature Methods 10, no. 8 (2013): 781–787, 10.1038/nmeth.2524. 23793239 PMC4071061

[25] L. P. Ong , J. Bargehr , V. R. Knight‐Schrijver , et al., “Epicardially Secreted Fibronectin Drives Cardiomyocyte Maturation in 3D‐Engineered Heart Tissues,” Stem Cell Reports 18, no. 4 (2023): 936–951, 10.1016/j.stemcr.2023.03.002. 37001515 PMC10147941

[26] A. Hansen , A. Eder , M. Bönstrup , et al., “Development of a Drug Screening Platform Based on Engineered Heart Tissue,” Circulation Research 107, no. 1 (2010): 35–44, 10.1161/CIRCRESAHA.109.211458. 20448218

[27] E. Ogawa , Y. Saito , M. Harada , et al., “Outside‐in Signalling of Fibronectin Stimulates Cardiomyocyte Hypertrophy in Cultured Neonatal Rat Ventricular Myocytes,” Journal of Molecular and Cellular Cardiology 32, no. 5 (2000): 765–776, 10.1006/jmcc.2000.1119. 10775482

[28] W. J. De Lange , E. T. Farrell , C. R. Kreitzer , et al., “Human iPSC‐Engineered Cardiac Tissue Platform Faithfully Models Important Cardiac Physiology,” American Journal of Physiology‐Heart and Circulatory Physiology 320, no. 4 (2021): H1670–H1686, 10.1152/ajpheart.00941.2020. 33606581 PMC8260387

[29] G. Conant , S. Ahadian , Y. Zhao , and M. Radisic , “Kinase Inhibitor Screening Using Artificial Neural Networks and Engineered Cardiac Biowires,” Scientific Reports 7, no. 1 (2017): 11807, 10.1038/s41598-017-12048-5. 28924210 PMC5603510

[30] S. Trujillo , C. Gonzalez‐Garcia , P. Rico , et al., “Engineered 3D Hydrogels With Full‐Length Fibronectin That Sequester and Present Growth Factors,” Biomaterials 252 (2020): 120104, 10.1016/j.biomaterials.2020.120104. 32422492

[31] O. Dobre , M. A. G. Oliva , G. Ciccone , et al., “A Hydrogel Platform That Incorporates Laminin Isoforms for Efficient Presentation of Growth Factors—Neural Growth and Osteogenesis,” Advanced Functional Materials 31, no. 21 (2021): 2010225, 10.1002/adfm.202010225.

[32] L. Sala , B. J. Van Meer , L. G. J. Tertoolen , et al., “Musclemotion: A Versatile Open Software Tool to Quantify Cardiomyocyte and Cardiac Muscle Contraction In Vitro and In Vivo,” Circulation Research 122 (2018): 3, 10.1161/CIRCRESAHA.117.312067. PMC5805275 29282212

[33] A. D. S. Costa , P. Mortensen , M. P. Hortigon‐Vinagre , et al., “Electrophysiology of hiPSC‐Cardiomyocytes Co‐Cultured With HEK Cells Expressing the Inward Rectifier Channel,” International Journal of Molecular Sciences 22, no. 12 (2021): 6621, 10.3390/ijms22126621. 34205607 PMC8235371

[34] M. P. Hortigon‐Vinagre , V. Zamora , F. L. Burton , J. Green , G. A. Gintant , and G. L. Smith , “The Use of Ratiometric Fluorescence Measurements of the Voltage Sensitive Dye Di‐4‐ANEPPS to Examine Action Potential Characteristics and Drug Effects on Human Induced Pluripotent Stem Cell‐Derived Cardiomyocytes,” Toxicological Sciences 154, no. 2 (2016): 320–331, 10.1093/toxsci/kfw171. 27621282 PMC5139069

[35] M. P. Hortigon‐Vinagre , V. Zamora , F. L. Burton , and G. L. Smith , “The Use of Voltage Sensitive Dye Di‐4‐ANEPPS and Video‐Based Contractility Measurements to Assess Drug Effects on Excitation–Contraction Coupling in Human‐Induced Pluripotent Stem Cell–Derived Cardiomyocytes,” Journal of Cardiovascular Pharmacology 77, no. 3 (2021): 280–290, 10.1097/FJC.0000000000000937. 33109927

[36] A. Sharma , W. L. McKeithan , R. Serrano , et al., “Use of Human Induced Pluripotent Stem Cell–Derived Cardiomyocytes to Assess Drug Cardiotoxicity,” Nature Protocols 13, no. 12 (2018): 3018–3041, 10.1038/s41596-018-0076-8. 30413796 PMC6502639

[37] N. Bildyug , “Extracellular Matrix in Regulation of Contractile System in Cardiomyocytes,” International Journal of Molecular Sciences 20, no. 20 (2019): 5054, 10.3390/ijms20205054. 31614676 PMC6834325

[38] S. Querceto , R. Santoro , A. Gowran , et al., “The Harder the Climb the Better the View: The Impact of Substrate Stiffness on Cardiomyocyte Fate,” Journal of Molecular and Cellular Cardiology 166 (2022): 36–49, 10.1016/j.yjmcc.2022.02.001. 35139328 PMC11270945

[39] M. Salmerón‐Sánchez and M. J. Dalby , “Synergistic Growth Factor Microenvironments,” Chemical Communications 52, no. 91 (2016): 13327–13336, 10.1039/c6cc06888j. 27722261

[40] W. H. Ziegler , R. C. Liddington , and D. R. Critchley , “The Structure and Regulation of Vinculin,” Trends in Cell Biology 16, no. 9 (2006): 453–460, 10.1016/j.tcb.2006.07.004. 16893648

[41] L. Guo , S. Eldridge , M. Furniss , J. Mussio , and M. Davis , “Use of Human Induced Pluripotent Stem Cell–Derived Cardiomyocytes (hiPSC‐CMs) to Monitor Compound Effects on Cardiac Myocyte Signaling Pathways,” Current Protocols in Chemical Biology 7, no. 3 (2015): 141–185, 10.1002/9780470559277.ch150035. 26331525 PMC4568555

[42] L. Matsuuchi and C. C. Naus , “Gap Junction Proteins on the Move: Connexins, the Cytoskeleton and Migration,” Biochimica Et Biophysica Acta (BBA)—Biomembranes 1828, no. 1 (2013): 94–108, 10.1016/j.bbamem.2012.05.014. 22613178

[43] S. Appukuttan , K. L. Brain , and R. Manchanda , “Effect of Variations in Gap Junctional Coupling on the Frequency of Oscillatory Action Potentials in a Smooth Muscle Syncytium,” Frontiers in Physiology 12 (2021): 655225, 10.3389/fphys.2021.655225. 34658901 PMC8517141

[44] S. H. Vermij , H. Abriel , and T. A. B. van Veen , “Refining the Molecular Organization of the Cardiac Intercalated Disc,” Cardiovascular Research 113, no. 3 (2017): 259–275, 10.1093/cvr/cvw259. 28069669

[45] Drugbank, Nifedipine , available from: https://go.drugbank.com/drugs/DB01115.

[46] G. Thomas , M. Chung , and C. J. A. D. Cohen , “A Dihydropyridine (Bay k 8644) that Enhances Calcium Currents in Guinea Pig and Calf Myocardial Cells. A new type of Positive Inotropic Agent,” Circulation Research 56, no. 1 (1985): 87–96, 10.1161/01.RES.56.1.87. 2578336

[47] AG Scientific. Thapsigargin, available from: https://agscientific.com/products/inhibitors/protein‐inhibitors/atpase‐inhibitors/thapsigargin‐1‐mg.html?srsltid=AfmBOoqy9hDGobhfahVHlInECOmv4Zo_RwLr0P7mpvUL_XfIiY.

[48] M. P. Hortigon‐Vinagre , V. Zamora , F. L. Burton , and G. L. Smith , “The Use of Voltage Sensitive Dye Di‐4‐ANEPPS and Video‐Based Contractility Measurements to Assess Drug Effects on Excitation–Contraction Coupling in Human‐Induced Pluripotent Stem Cell–Derived Cardiomyocytes,” Journal of Cardiovascular Pharmacology 77, no. 3 (2021): 280–290, 10.1097/FJC.0000000000000937. 33109927

[49] H. Zeng , J. Wang , H. Clouse , and A. Lagrutta , “Unveiling the Lack of Inotropic Response of Human Induced Pluripotent Stem Cell‐Derived Cardiomyocytes to Isoproterenol by Chronic External Stimulation,” Applied In Vitro Toxicology 6 (2020): 65–71, 10.1089/aivt.2020.0002.

[50] D. Fixler , R. Tirosh , T. Zinman , A. Shainberg , and M. Deutsch , “Differential Aspects in Ratio Measurements of [Ca2+]i Relaxation in Cardiomyocyte Contraction Following Various Drug Treatments,” Cell Calcium 31, no. 6 (2002): 279–287, 10.1016/S0143-4160(02)00056-8. 12098217

[51] J. Layland and J. C. Kentish , “Myofilament‐based relaxant effect of isoprenaline revealed During work‐loop contractions in rat cardiac trabeculae,” The Journal of Physiology 544, no. 1 (2002): 171–182, 10.1113/jphysiol.2002.022855. 12356890 PMC2290578

[52] H. Yang , Y. Yang , F. N. Kiskin , M. Shen , and J. Z. Zhang , “Recent Advances in Regulating the Proliferation or Maturation of Human‐Induced Pluripotent Stem Cell‐Derived Cardiomyocytes,” Stem Cell Research & Therapy 14, no. 1 (2023): 228, 10.1186/s13287-023-03470-w. 37649113 PMC10469435

[53] M. Goßmann , R. Frotscher , P. Linder , et al., “Mechano‐Pharmacological Characterization of Cardiomyocytes Derived From Human Induced Pluripotent Stem Cells,” Cellular Physiology and Biochemistry 38, no. 3 (2016): 1182–1198, 10.1159/000443124. 26983082

[54] S. Sicouri , K. W. Timothy , A. C. Zygmunt , et al., “Cellular Basis for the Electrocardiographic and Arrhythmic Manifestations of Timothy Syndrome: Effects of Ranolazine,” Heart Rhythm 4 (2007): 638–647, 10.1016/j.hrthm.2006.12.046. 17467634 PMC1951535

[55] L. Guo , J.‐Y. Qian , R. Abrams , et al., “The Electrophysiological Effects of Cardiac Glycosides in Human iPSC‐Derived Cardiomyocytes and in Guinea Pig Isolated Hearts,” Cellular Physiology and Biochemistry 27, no. 5 (2011): 453–462, 10.1159/000329966. 21691062

[56] E. Huethorst , P. Mortensen , R. D. Simitev , et al., “Conventional Rigid 2D Substrates Cause Complex Contractile Signals In Monolayers Of Human Induced Pluripotent Stem Cell‐Derived Cardiomyocytes,” The Journal of Physiology 600, no. 3 (2022): 483–507, 10.1113/jp282228. 34761809 PMC9299844

