# Supplementary material for: Mechanical and Electrical Phenotype of hiPSC‐Cardiomyocytes on Fibronectin‐Based Hydrogels
Source: Adv Healthc Mater. 2025 Nov 29;15(27):e01595. doi: 10.1002/adhm.202501595 (PMC13378479; doi:10.1002/adhm.202501595)
Supplement: Supplementary file 1 — Supporting file: adhm70519‐sup‐0001‐SuppMat.docx [file ADHM-15-0-s001.docx]

**Mechanical and electrical phenotype of hiPSC-cardiomyocytes on fibronectin-based hydrogels**

Ana Da Silva Costa^1,2^, Lineta Stonkute, Sara Trujillo^3^, Mariana Azevedo Gonzalez Oliva^3,4^, Francis Burton^1^, Matthew J. Dalby^3^, Oana Dobre^3^, Godfrey Smith^1*^, and Manuel Salmeron-Sanchez^3,4,5*^

1. School of Cardiovascular and Metabolic Health, University of Glasgow, UK

2. MVLS Graduate School, University of Glasgow, UK

3. Centre for the Cellular Microenvironment, University of Glasgow, UK

4. Institute for Bioengineering of Catalonia (IBEC), The Barcelona Institute for Science and Technology (BIST), 08028 Barcelona, Spain

5. Institució Catalana de Recerca i Estudis Avançats (ICREA), Barcelona, Spain

To whom correspondence should be addressed

[Godfrey.Smith@glasgow.ac.uk](mailto:Godfrey.Smith@glasgow.ac.uk)

[Manuel.Salmeron-Sanchez@glasgow.ac.uk](mailto:Manuel.Salmeron-Sanchez@glasgow.ac.uk) | [msalmeron@ibecbarcelona.eu](mailto:msalmeron@ibecbarcelona.eu)

**SUPPLEMENTARY MATERIAL**

# Appendix

## Macro FIJI for immunofluorescence analysis:

run("Subtract Background...", "rolling=30");

run("Enhance Local Contrast (CLAHE)", "blocksize=15 histogram=256 maximum=6 mask=*None*");

run("Exp");

run("Threshold...");

waitForUser("Set the threshold and press OK, or cancel to exit macro");

setOption("BlackBackground", false);

run("Convert to Mask");

run("Invert");

run("Set Scale...", "distance=1 known=0.12153 unit=um");

run("Set Measurements...", "area mean fit redirect=None decimal=3");

run("Analyze Particles...", "size=0.75-Infinity circularity=0.00-0.99 display clear include summarize");

A.


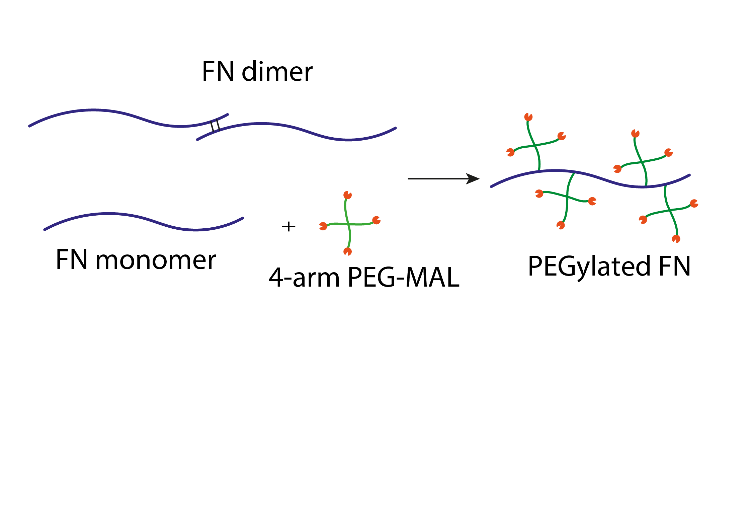


B.


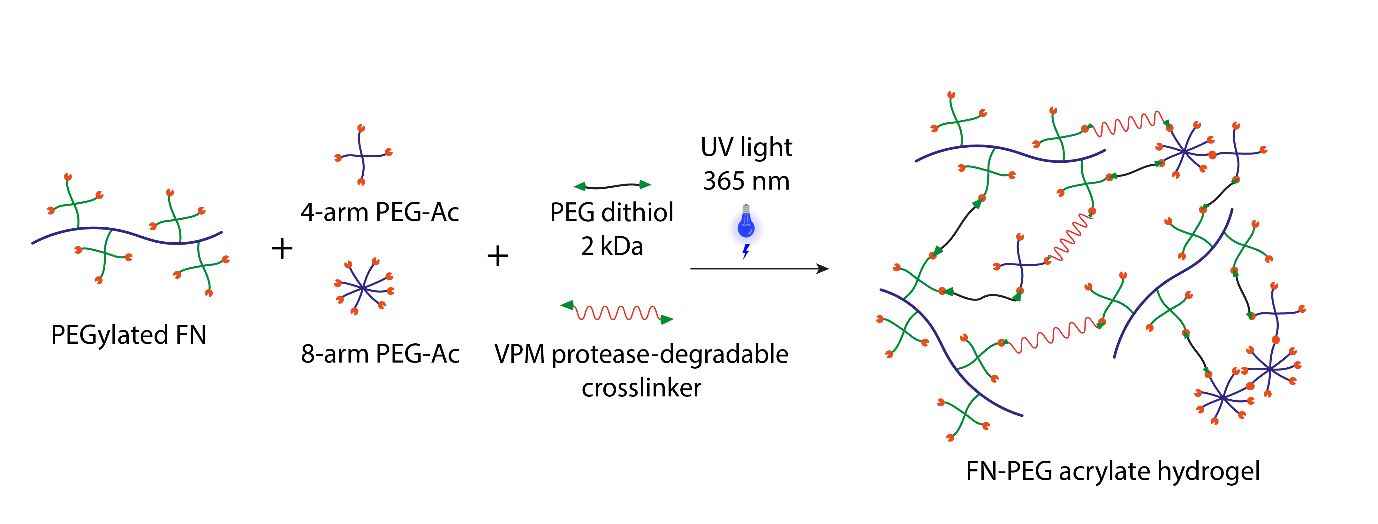


*Supplementary Figure 1 – Fibronectin hydrogels chemistry. A) FN/PEG gel formulation schematic drawing, where the first step presented is PEGylation, the reaction used to functionalise FN molecules with MAls at different ratios, followed by photopolymerisation, reaction used to crosslink the FN-PEG gel*


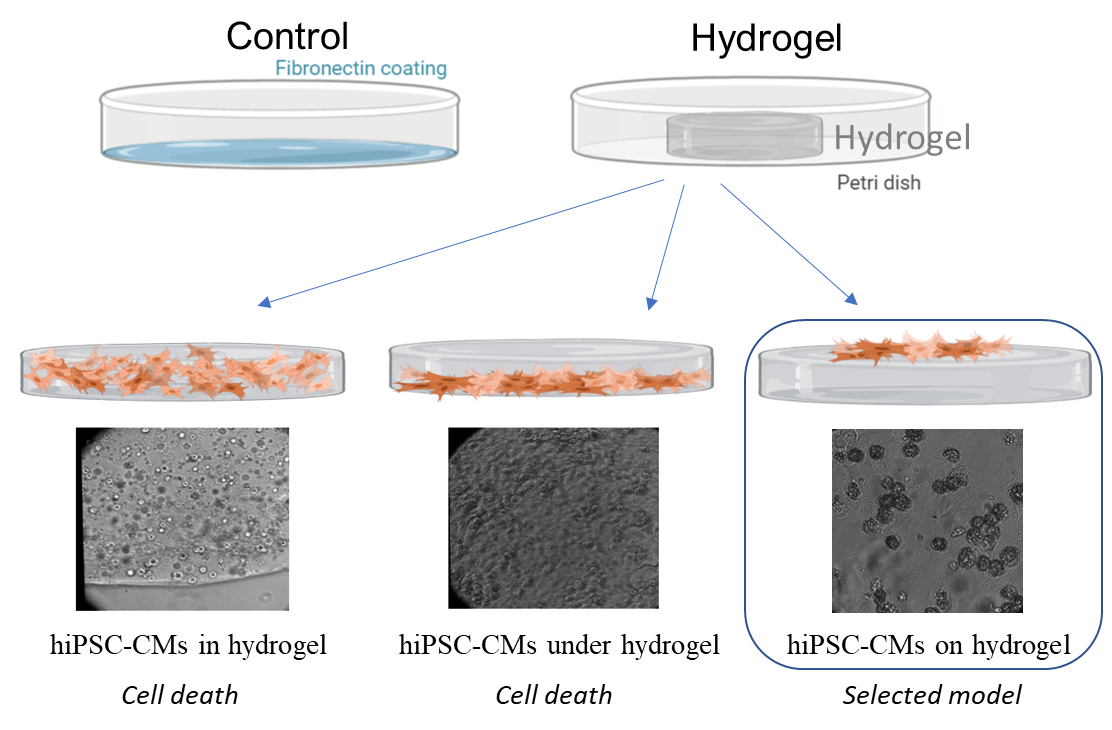


*Supplementary Figure 2 – Initial experimental work investigated the effects of culturing hiPSC-CMs in a 2D model by imbedding in a hydrogel and curating using a UV source, or by culturing a monolayer and curating a hydrogel on top of cell layer. Both methods led to quiescence and the experimental protocol was modified to seeding hiPSC-CMs on previously curated hydrogels.*


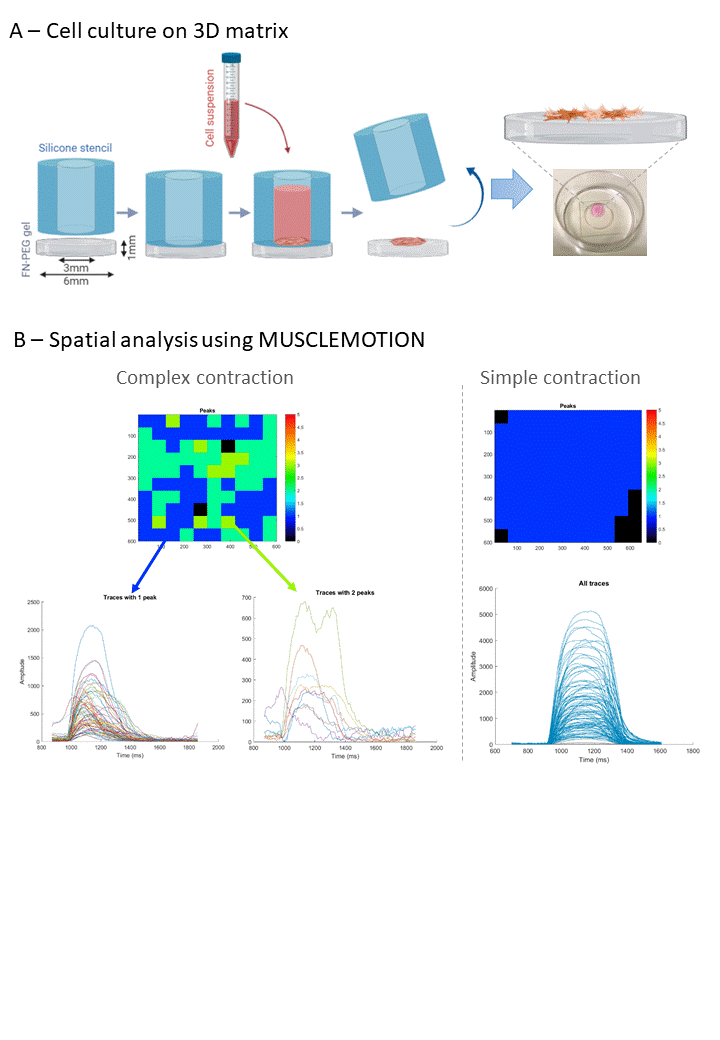


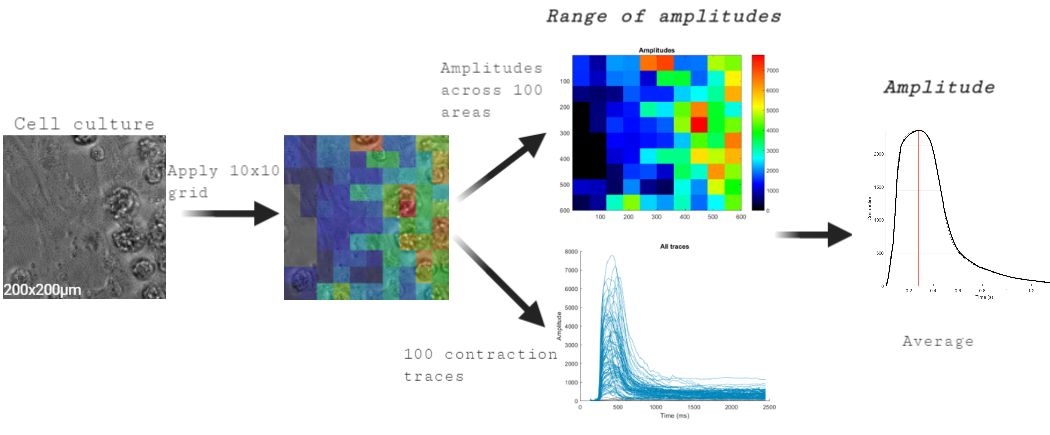


*Supplementary Figure 3 - Analysis of contraction. Contraction profile - Spatial analysis using MUSCLEMOTION. A 10x10 grid is applied to the cell motion video, which provides the traces for each of the 100 areas. Complex contraction occurs when some of the contraction traces display more than one peak, whereas simple contraction shows only single peaks.*
